# Thrombosed Large Distal Posterior Inferior Cerebellar Artery Aneurysm Mimicking an Infratentorial Ependymoma

**DOI:** 10.1155/2014/435953

**Published:** 2014-02-11

**Authors:** Peter Yat Ming Woo, Natalie Man Wai Ko, Kwong Yau Chan

**Affiliations:** Department of Neurosurgery, Kwong Wah Hospital, 25 Waterloo Road, Hong Kong

## Abstract

Large or giant intracranial aneurysms can simulate brain tumors clinically and radiologically by virtue of their progressive mass effect. Unlike aneurysms from alternative locations, those arising from the distal posterior inferior cerebellar artery (PICA) are uncommon. We report a patient who experienced progressive hemiparesis with magnetic resonance imaging findings suggestive of an infratentorial ependymoma. Intraoperatively, a thrombosed large aneurysm of the distal PICA was unexpectedly encountered. The aneurysm was clipped and the patient did not develop any permanent neurological deficit. This case illustrates the radiological nuances of large aneurysms and infratentorial ependymomas. Three-dimensional contrast-enhanced magnetic resonance angiography can be falsely negative and the importance of the “target” sign is emphasized. One should be cognizant of this possible diagnosis for patients with midline fourth ventricular lesions in order to reduce surgical risk.

## 1. Introduction

Aneurysms arising from the posterior inferior cerebellar artery (PICA) are uncommon and constitute 0.5 to 3.0% of all intracranial aneurysms [1]. While most arise at its origin from the vertebral artery, distal PICA aneurysms are less frequently encountered with an incidence of 0.3 to 1.4% [1–3]. Large aneurysms, defined as a diameter equal to or greater than 15 mm, arising from the distal PICA are rare [2, 4]. Half of these lesions are thrombosed on presentation and can imitate a posterior fossa neoplasm [5]. Occasionally the neurosurgeon is confronted with this unexpected finding intraoperatively and is required to immediately alter surgical strategies. Although posterior circulation aneurysms have been described to mimic tumors on computed tomography (CT) imaging since the 1970s, the occurrence of such has diminished with the advent of magnetic resonance imaging (MRI) and MR angiography (MRA) [5–12]. We report a patient with a partially thrombosed large distal PICA aneurysm that simulated an infratentorial ependymoma which was only discovered intraoperatively. The radiological features of these pseudotumor aneurysms are discussed.

## 2. Clinical Presentation

A 79-year-old woman, with a history of hypertension, presented with dizziness and progressive left side weakness for two months. There was neither headache nor symptoms to suggest raised intracranial pressure. She found it increasingly difficult to walk and required assistance.

Physical examination revealed left hemiparesis of medical research council (MRC) power grade 4/5. Deep tendon reflexes were equivocal and muscle tone was normal. Cranial nerve palsy, cerebellar signs, and papilloedema were not detected.

**Figure 1:**
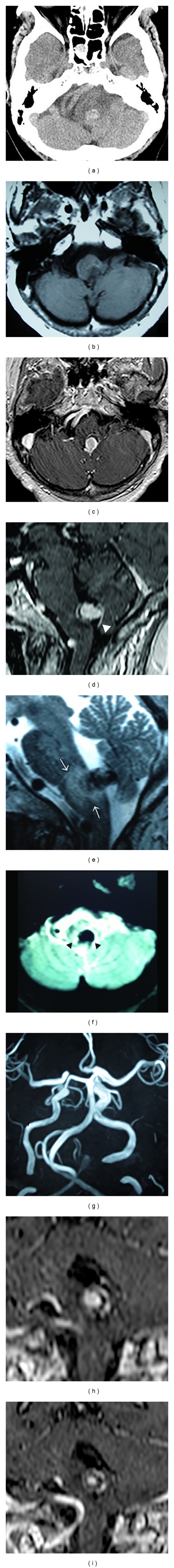
Noncontrast enhanced CT brain showing a hyperdense midline lesion of the posterior fossa ((a), axial). T1W MRI sequences depict a hypointense lesion, apparently intra-axial, arising from the floor of the fourth ventricle compressing against the superior medulla ((b), axial). The lesion demonstrates gadolinium contrast enhancement and is abutting the PICA, white arrowhead ((c), T1W contrast, axial; (d), sagittal). T2W MRI revealing significant perilesional medullary edema, white arrows ((e), sagittal). SWI sequence showing blooming artifacts within the lesion and its proximity to the PICA, black arrowheads ((f), axial). Three-dimensional reconstructed contrast-enhanced MRA showing no detectable vascular lesion (g). The “target” sign is observed only in the coronal contrast T1W images ((h), (i)).

Computed tomography revealed a midline hyperdense posterior fossa lesion that displaced the fourth ventricle and compressed the pontine-medullary junction of the brainstem (Figure 1(a)). There was also mild obstructive hydrocephalus. A noncontrast enhanced T1-weighted (T1W) and T2-weighted MRI depicted a hypointense circumscribed 20 mm lesion that arose from the fourth ventricular floor causing significant mass effect on the superior medulla (Figures 1(b) and 1(e)). There was heterogeneous gadolinium contrast enhancement (Figures 1(c) and 1(d)). Susceptibility weighted imaging (SWI) demonstrated “blooming” effects indicating calcifications and blood products within the lesion. Both contrast T1W and SWI sequences showed that it was in close proximity to the telovelotonsillar segment of the PICA (Figure 1(f)). Three-dimensional (3D) contrast-enhanced MRA did not show any vascular abnormalities (Figure 1(g)). Upon further multiplanar assessment, coronal imaging showed intralesional and rim contrast enhancement that resembled a “target” sign (Figures 1(h) and 1(i)). The initial diagnosis was an infratentorial ependymoma given the lesion's midline location, contrast enhancement pattern, and evidence of calcifications with hemorrhage.

**Figure 2:**
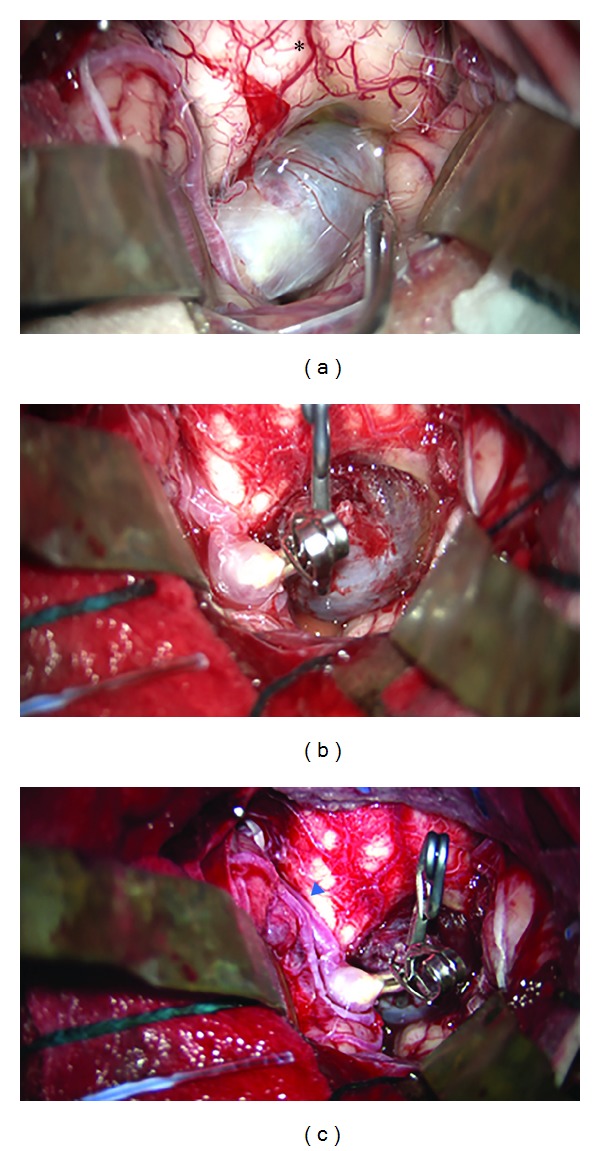
Operative photographs showing a well-encapsulated lesion upon lateral retraction of the cerebellar hemispheres ((a); asterisk, inferior medulla). Further dissection revealed a large aneurysm of the left distal PICA telovelotonsillary segment ((b), (c); blue arrowhead, PICA). Subsequent clipping of the aneurysm neck was performed with preservation of the PICA trunk ((c); blue arrowhead, PICA).

**Figure 3:**
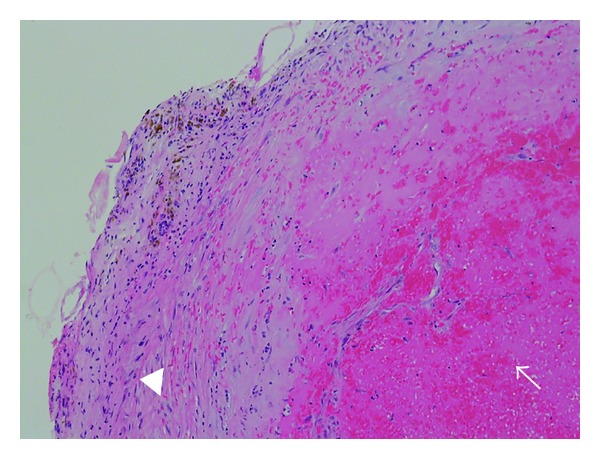
Photomicrograph, hematoxylin and eosin stain, displaying a fibrocollagenous aneurysm wall with hemosiderin deposition (arrowhead) and intraluminal fibrin clot with early organization (arrow).

A suboccipital craniotomy was performed and the telovelar approach was planned for tumor excision. Exploration of the cerebellomedullary fissure revealed a fibrous encapsulated 3 cm extra-axial lesion that indented the floor of the fourth ventricle. Upon microsurgical dissection, it transpired that the lesion was a saccular aneurysm arising from the telovelotonsillar segment of the left PICA with no perforator incorporation (Figure 2(a)). Clipping of the aneurysm was conducted with preservation of the parent artery and the dome was excised (Figures 2(b) and 2(c)). Histological examination confirmed the lesion to be a thrombosed aneurysm (Figure 3). Postoperatively, the patient developed transient vocal cord palsy and recovered fully from her hemiparesis with no evidence of lateral medullary syndrome.

## 3. Discussion

The posterior inferior cerebellar artery has five segments: (1) the anterior medullary segment; (2) the lateral medullary segment; (3) the telovelomedullary segment containing the caudal loop ending at the cerebellar tonsils; (4) the telovelotonsillar segment from the medial aspect of the tonsils to the roof of the fourth ventricle containing its cranial loop; and (5) the cortical segment that supplies the cerebellar vermis and hemispheres [13]. According to Lewis's surgical-anatomical classification, the distal PICA is defined as the telovelotonsillar and cortical segments since they do not give rise to brainstem perforator arteries [2]. The regions perfused by the distal PICA share intricate arteriolar anastomoses with contributions from the contralateral PICA and anterior inferior cerebellar and superior cerebellar arteries [1]. For these reasons if the artery must be occluded at this level the risk of significant neurological deficit is minimal [2].

Distal PICA aneurysms are uncommon and their pathogenesis is a matter of debate [1–3]. While most intracranial aneurysms are located at arterial bifurcations, distal PICA lesions arise predominantly from the arterial side wall of the telovelotonsillary segment. It was proposed that increased hemodynamic wall shear stress induced by the hairpin curvature of the cranial loop within this segment is responsible for aneurysm formation [2, 3, 14].

Our patient demonstrates how a large partially thrombosed distal PICA aneurysm can mimic an infratentorial ependymoma both clinically and radiologically. Most large distal PICA aneurysms rarely rupture and seldom cause thromboembolic events due to the rich anastomotic arterial supply of the inferior cerebellum. Their presentation is similar to posterior fossa neoplasms inducing chronic brainstem or cerebellar compression [14]. Deficits such as truncal ataxia, hemiparesis, and intractable hiccups have been reported [2, 15].

Radiologically infratentorial ependymomas can share common characteristics with partially thrombosed distal PICA aneurysms. Both are predominantly midline lesions located near the fourth ventricle. They can appear iso- or hypointense on T1W imaging and elicit heterogeneous contrast enhancement with significant perilesional vasogenic edema. Almost half of all ependymomas demonstrate T1- and T2-weighted signal heterogeneity due to areas of necrosis, cystic change, repeated hemorrhage, or calcifications [16]. Lamellar organization of subacute to chronic hemorrhagic components within an aneurysm could lead to similar features [13]. Both lesions can exhibit calcifications or hemorrhage on SWI. This case illustrates that 3D contrast-enhanced MRA can fail to identify thrombosed large aneurysms and the clinician should be aware of this limitation. Even if catheter angiography was performed, completely thrombosed aneurysms or those with minimal flow within its core are notorious for evading detection [5, 8–10].

The most significant radiological feature for this patient was the presence of the “target” sign. Best demonstrated on gadolinium contrast T1W imaging, it represents enhancement of the aneurysm wall as well as within the cavity of a partially thrombosed aneurysm [5]. This is the first reported case exhibiting the “target” sign on MRI as the sole distinguishing feature differentiating an infratentorial ependymoma and a partially thrombosed large distal PICA aneurysm. Retrospective evaluation of the MRI revealed the presence of this sign only on the coronal plane images. Another supportive radiological manifestation would be the presence of peripheral concentric calcifications on CT, but this was not observed in this case. Nevertheless, the older age of the patient should alert the clinician to entertain the possibility of a large or giant, defined as a diameter equal to or greater than 25 mm, aneurysm since infratentorial ependymomas predominate in children with a mean age of clinical manifestation of six years [4, 17].

Clipping is an effective means of treating distal PICA aneurysms, as they are often superficial and surgically easily accessible. Most patients have good functional outcomes as described in several operative series [1–3, 18, 19].

The clinician must be aware of the limitations of 3D MRA in identifying thrombosed aneurysms. We recommend meticulous multiplanar evaluation of contrast T1-weighted MRI for the “target” sign in older patients with midline fourth ventricular mass lesions. Catheter angiography is recommended in suspected cases and if they remain undetectable one should be cognizant of this possible diagnosis when surgically treating such lesions.
